# Molecular Characterization of Carbapenem and Colistin Resistance in *Klebsiella pneumoniae* Isolates Obtained from Clinical Samples at a University Hospital Center in Algeria

**DOI:** 10.3390/microorganisms12101942

**Published:** 2024-09-25

**Authors:** Riyane Rihane, Abla Hecini-Hannachi, Chafia Bentchouala, Kaddour Benlabed, Seydina M. Diene

**Affiliations:** 1 Molecular and Cellular Biology Laboratory, University of Mentouri Brothers Constantine 1, Constantine 25000, Algeria; 2 Department of Medicine, Faculty of Medicine, University of Salah Boubnider Constantine 3, Constantine 25000, Algeria; c.bentchouala@yahoo.fr (C.B.); kbenlabed@yahoo.fr (K.B.); 3 Bacteriology Laboratory, Benbadis University Hospital, Constantine 25000, Algeria; 4 Microbes Evolution Phylogeny and Infections (MEPHI), Institut de Recherche pour le Développement (IRD), Assistance Publique-Hopitaux de Marseille (AP-HM), IHU-Méditerranée Infection, Faculté de Pharmacie, Aix-Marseille University, 13385 Marseille, France; seydina.diene@univ-amu.fr

**Keywords:** *Klebsiella pneumoniae*, antibiotic, resistance genes, carbapenem, colistin

## Abstract

The current study aimed to determine the molecular mechanisms of carbapenem and colistin resistance among the clinical isolates of *Klebsiella pneumoniae* from hospitalized patients admitted to a university hospital in Eastern Algeria. In total, 124 non-duplicate isolates of *K. pneumoniae* were collected from September 2018 to April 2019. Bacterial identification was performed using MALDI-TOF MS. The presence of extended spectrum β-lactamase (ESBL) genes, carbapenemase genes, chromosomal mutation and *mcr* genes in colistin-resistant *K. pneumoniae* were evaluated by PCR. ESBLs represented a rate of 49.1% and harbored *bla*_CTX-M_, *bla*_TEM_ and *bla*_SHV_ genes. Concerning carbapenems, 12 strains (9.6%) were resistant to ertapenem (MIC: 1–32 μg/mL), of which one strain (0.8%) was also resistant to imipenem (MIC: 32 μg/mL). Among these strains, nine (75%) harbored *bla*_OXA-48_ gene. Seven strains (5.6%) expressed resistance to colistin (MIC: 2–32 μg/mL), of which two harbored *mcr-8* and *mgrB* genes simultaneously. The existence of a double resistance to colistin in the same strain is new in Algeria, and this could raise concerns about the increase in levels of resistance to this antibiotic (MIC: 32 μg/mL). The *mgrB* gene alone was observed in five isolates (71.4%), including two strains harboring *bla*_OXA-48_. This is the first report revealing the presence of *K. pneumoniae* strains carrying the *bla*_OXA-48_ gene as well as a mutation in the *mgrB* gene. Large-scale surveillance and effective infection control measures are also urgently needed to prevent the outbreak of various carbapenem- and colistin-resistant isolates.

## 1. Introduction

Gram-negative bacterial (GNB) resistance to antimicrobials is increasing worldwide. *Klebsiella pneumoniae* is one of the major causes of nosocomial infections that can carry many antibiotic resistance genes such as extended-spectrum β-lactamases (ESBLs), and because of the production of these enzymes, the use of third and fourth generation cephalosporins is restricted [1]. Carbapenem antibiotics are recommended as one of the last lines of therapy for multidrug-resistant (MDR) strains of *K. pneumoniae*. However, increasing the rate of resistance to carbapenems has complicated the treatment process and led to untreatable hospital infections [2]. 

The mortality rate associated with carbapenem-resistant *K pneumoniae* (CR-Kp) infections is alarmingly high, with risk factors including ICU stays and comorbid conditions [3]. CR-Kp poses a significant threat due to its complex resistance mechanisms and widespread dissemination. CR-Kp exhibits resistance through several mechanisms, with a key one being the production of carbapenemases, which are classified into Ambler classes A (e.g., *K pneumoniae* carbapenemase, KPC), B (e.g., New Delhi metallo-β-lactamases, NDM), and D (e.g., OXA-48-like carbapenemases). These enzymes hydrolyze carbapenems and are typically encoded on mobile genetic elements (MGEs), facilitating their horizontal transfer [4,5,6,7]. KPC-producing strains exhibit high clonal expansion and mobile gene transfer, which complicates control efforts as these strains can persist in human reservoirs and form biofilms that resist hospital disinfection protocols [4,5,6]. The spread of carbapenem resistance has been linked to IncC plasmids, which have a broad host range and often carry carbapenemase genes. These plasmids contribute to the regional and global spread of resistance [8]. In recent studies, IncC plasmids have been increasingly reported in carbapenem-resistant bacterial isolates across Asia, highlighting the need for ongoing surveillance [8]. Resistance mechanisms also involve efflux pumps and porin loss, in addition to enzymatic activity [9]. 

The rising incidence of carbapenem resistance in the *K. pneumoniae* complex, coupled with the absence of new antibiotic classes effective against GNB, has significantly restricted treatment options for carbapenem-resistant strains of this complex. As a result, there has been a renewed reliance on colistin for managing infections caused by these resistant *K. pneumoniae* strains [10]. Colistin is now recommended as the last choice for the treatment of MDR GNB, especially carbapenem-resistant *Enterobacteriaceae* (CRE) infections [11]. The recent increase in the use of colistin in clinical practice, accompanied by its unbridled use in agriculture, has contributed to the rapid dissemination of resistance [12]. Polymyxin resistance was thought to occur only through chromosomal mutations between the genes regulating pmrA/B and phoP/Q binary systems or mutations in the *mgrB* gene [13,14], leading to amino acid changes, insertions, and deletions in the proteins produced by these genes [10]. In *K. pneumoniae*, a common mechanism of colistin resistance involves the inactivation of *mgrB*, which encodes a negative regulator of the *PhoPQ* two-component regulatory system. This inactivation often occurs due to the insertion of an insertion sequence (IS) element or mutations that create premature stop codons [10,15], but the recent discovery of the plasmid-dependent *mcr-1* revealed another form of resistance to colistin. This gene is based on moving genetic elements and due to horizontal gene transfer; it has caused resistance and has therefore spread throughout the world. The acquisition of mobile genetic elements containing genes from the mcr family can also result in modifications to lipids [16,17]. Following the discovery of *mcr-1*, other genes such as *mcr-2* and *mcr*-9 have been reported [16], as well as the recently identified *mcr-10* [18]. However, there have been few reports of the presence of *mcr-1*, *mcr-3*, *mcr-7*, and *mcr-8* in *K. pneumoniae*, with these variants occurring at relatively low prevalence [19]. All MCR proteins function as phosphoethanolamine (PEtN) transferases, which facilitate the addition of PEtN to lipid A. This modification decreases the negative charge of lipopolysaccharides (LPSs) by altering lipid A’s structure, thereby reducing colistin binding and leading to colistin resistance [20]. The spread of this plasmid-mediated resistance from animals—where colistin has been used for years as a therapeutic agent or food additive—to humans via horizontal gene transfer has been suggested. Mcr gene-carrying *Enterobacteriaceae* are found more frequently on hospital surfaces than in clinical isolates, indicating the plasmid’s ability to spread not only in vitro but also among key human pathogens. The persistent contamination of hospital surfaces may thus facilitate the spread of colistin resistance among GNB, potentially exacerbated by selective pressures from some antiseptics (e.g., chlorhexidine) [16].

Other mechanisms contributing to colistin resistance in *K. pneumoniae* include the increased expression of efflux pumps, alterations in lipopolysaccharide (LPS) production, the excessive production of capsular polysaccharides, and the production of colistinase [21,22]. Due to the lack of information about the resistance of clinical *K. pneumoniae* to carbapenem and colistin in Algeria, the main purpose of this study was to evaluate the antimicrobial resistance patterns and molecular mechanisms of carbapenem and colistin resistance among the clinical isolates of *K. pneumoniae* from hospitalized patients admitted to a university hospital in Eastern Algeria.

## 2. Materials and Methods

### 2.1. Study Design and Clinical Sample Collection

In this prospective and monocentric study conducted at the Benbadis University Hospital Centre in Constantine, Algeria, over seven months (September 2018–April 2019), 124 non-redundant *K pneumoniae* strains were isolated from hospitalized patients of all ages and both sexes. Strains were selected based on clinical relevance, with one strain per patient included to avoid redundancy. Isolates were obtained from various clinical samples, such as blood, urine, and respiratory secretions, and identified using standard microbiological methods. Our work has been approved by the ethics committee of Constantine Hospital to carry out this research. Our strains were identified after being cultivated and incubated for 24 h at 37 ± 1 °C on MacConkey agar (BioMerieux, Marcy l’Etoile, France), and the identification of each strain was performed by using the MALDI TOF MS (Microflex LT) spectrometer (Bruker Daltonics, Bremen, Germany) by testing a colony from a pure culture two times in order to confirm our identification.

### 2.2. Antimicrobial Susceptibility Testing

The antibiotic susceptibility testing of clinical specimens was performed using the disk diffusion method on Mueller–Hinton (MH) agar II (Becton Dickinson Bioscience, east Rutherford, new jersey) according to recommendations of the European Committee on Antimicrobial Susceptibility Testing (EUCAST) 2021. The following antibiotics disks were used to assess the susceptibility of *Enterobacteriaceae* isolates: amoxicillin (AMX20), amoxicillin + clavulanic acid (AMC30), cefepime (FEP 30), piperacillin + tazobactam (TZP 36), mecillinam (MEC 10), ceftriaxone (CRO30), ertapenem (ETP10), imipenem (IPM10), fosfomycin (FF200), nitrofurantoin (F100), trimethoprim + sulfamethoxazole (SXT25), amikacin (AK30), ciprofloxacin (CIP5), tetracycline (TET30), colistin (CS50) and gentamicin (GN10) (Sirscan, Montpellier, France) All isolates were screened for ESBL via the synergy test between a third-generation cephalosporin and clavulanate (CRO, AMC, FEP). For strains resistant to imipenem (IPM) and ertapenem (ETP), the minimum inhibitory concentrations (MICs) were determined using the Etest method (Biomerieux, Craponne, France). Additionally, a qualitative colorimetric test (the β CARBA test) was performed to detect the production of carbapenemase ^16^. Colistin MIC was performed using the microdilution method (the UMIC test). Susceptibility patterns were interpreted according to the EUCAST 2021 guidelines (www.eucast.org accessed on 14 March 2023) using the Interscience scan 4000 France.

### 2.3. Molecular Investigation of Antibiotic Resistance Genes

The presence of ESBL and carbapenemase resistance genes was determined by real-time PCR (RT-PCR) reactions. For this purpose, a colony of each culture of our bacterial isolates was taken and was resuspended in 200 µL in distilled sterile water (Biorad, Marnes-la-Coquette, France) and heated to 70 °C for 10 min and then agitated for about 1 min in order to extract the strains DNA. Our ready DNA fractions were directly used for RT-PCR and standard PCR reactions, using CFX OPUS 96 REAL TIME PCR SYSTEM (Biorad, Marnes-la-Coquette, France) and PCR SYSTEM (Bio-Rad). Our extracted DNA was stored at −20 °C for further experiments.

### 2.4. Primer and TaqMan Probes

The references and information about the already characterized primers and probes were obtained from the Jean-Marc Rolain laboratory [23], and the protocol followed for the RT-PCR was provided by the Jean-Marc Rolain laboratory and it was as follows: 10 µL of Master Mix Kit (Qiagen, Germantown, MD, USA), 1 µL of the Forward (F), 1 µL of the Reverse (R), and 1 µL of the Probe (P), 2 µL H_2_O (Qiagen, Germantown, MD, USA) added to 5 µL of our extracted DNA.

### 2.5. Multiplex Real-Time PCR

The following β-lactamases genes were targeted, *bla_TEM_*, *bla*_SHV_ and *bla*_CTX_-_M A B_, and carbapenemase *bla*_KPC_, *bla*_VIM_, *bla*_NDM_ and *bla*_OXA_-_23 24 48 58_, as well as colistin resistance genes *mcr-*1, *mcr-*2, *mcr-*3, *mcr-*5, and *mcr-*8. Our strains were firstly divided into 14 pools, and each one contained between 18 and 21 bacteria to determine the maximum number of positive individuals [23]; only positive pools were treated after separately in order to detect the presence of the resistance genes in each bacterium. The detection of the resistance genes was performed for all isolates using the Jean-Marc Rolain RT-PCR protocol. 

### 2.6. Standard PCR

A standard PCR followed by migration on agarose gel was performed on *mgrB* in order to detect the presence of chromosomal resistance among our colistin-resistant strains, then BigDye PCR and sequencing were performed according to the instructions provided by the manufacturer (Macherey-Nagel, Düren, Germany); the standard PCR for the *mgrB* gene was purified using a Millipore NucleoFast 96 PCR kit followed by gel electrophoresis. Sanger sequencing was then carried out using the BigDye Terminator Cycle sequencer kit from Applied Biosystems on an ABI 3130 automatic sequencer. ChromasPro 1.7 software (Technelysium Pty Ltd., Tewantin, Australia) was then used to construct and evaluate the resultant sequences.

### 2.7. Primers and Probes for PCR and qPCR

The primers and probes used for real-time PCR (Table 1) and standard PCR (Table 2) in this study are listed in the following tables. These were designed to target specific resistance genes and were validated using positive controls as referenced.

### 2.8. Statistical Analysis

The comparison of resistance in carbapenemase-producing *K. pneumoniae* isolates with sensitive isolates was analyzed by performing the Pearson chi-square test with Yates’ correction using R statistical software version Rx64-4.0.2 for Windows. The significance level was set at a *p-*value < 0.05.

## 3. Results

### 3.1. Distribution of Strains by Sample Source, Hospital Department, and Patient Demographics

**Figure 1:**
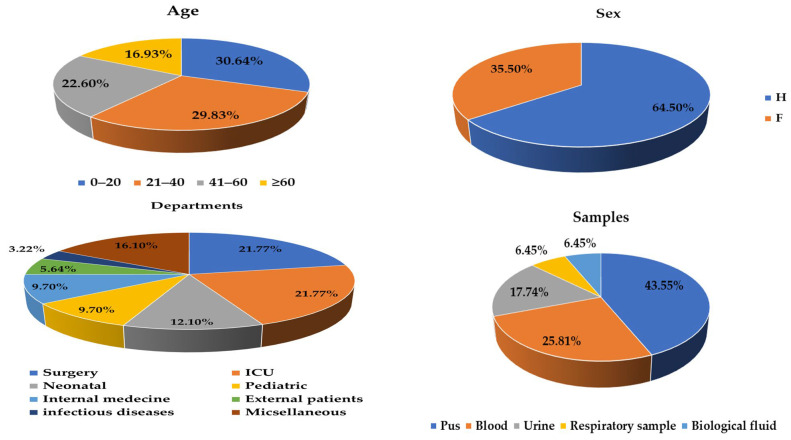
Distribution of *K. pneumoniae* clinical isolates according to: age, sex, department, clinical sample.

Moreover, 124 strains of *K. pneumoniae* were isolated in our study. Depending on the collection site, the clinical samples were divided into pus (43.5%, 54/124), blood (25.8%, 32/124), urine (17.7%, 22/124), respiratory samples and biological fluids (6.4%, 8/124 each). The surgical department and intensive care unit represented the highest isolate rates, with 21.7% of cases (27/124) each. Other services such as neonatology, pediatrics, internal medicine, infectious diseases and outpatients had lower rates, respectively (12%, 9.6%, 9.6%, 5.6% and 3.2%). Other services were identified in our study in very small proportions and grouped into miscellaneous (16%) (Figure 1). Most of our patients were men (64.5%, 80/124), with a sex ratio of 1.88. Depending on age, almost equal rates were found in the age groups, one going from 0 to 20 years (30.6%, 38/124) and the other whose age was between 21 and 40 years (29.8%, 37/124) (Figure 1); this rate was the highest among the rest of the age range, with a mean of 33 years and a median of 31 years (Figure 1).

### 3.2. Antimicrobial Susceptibility

**Figure 2:**
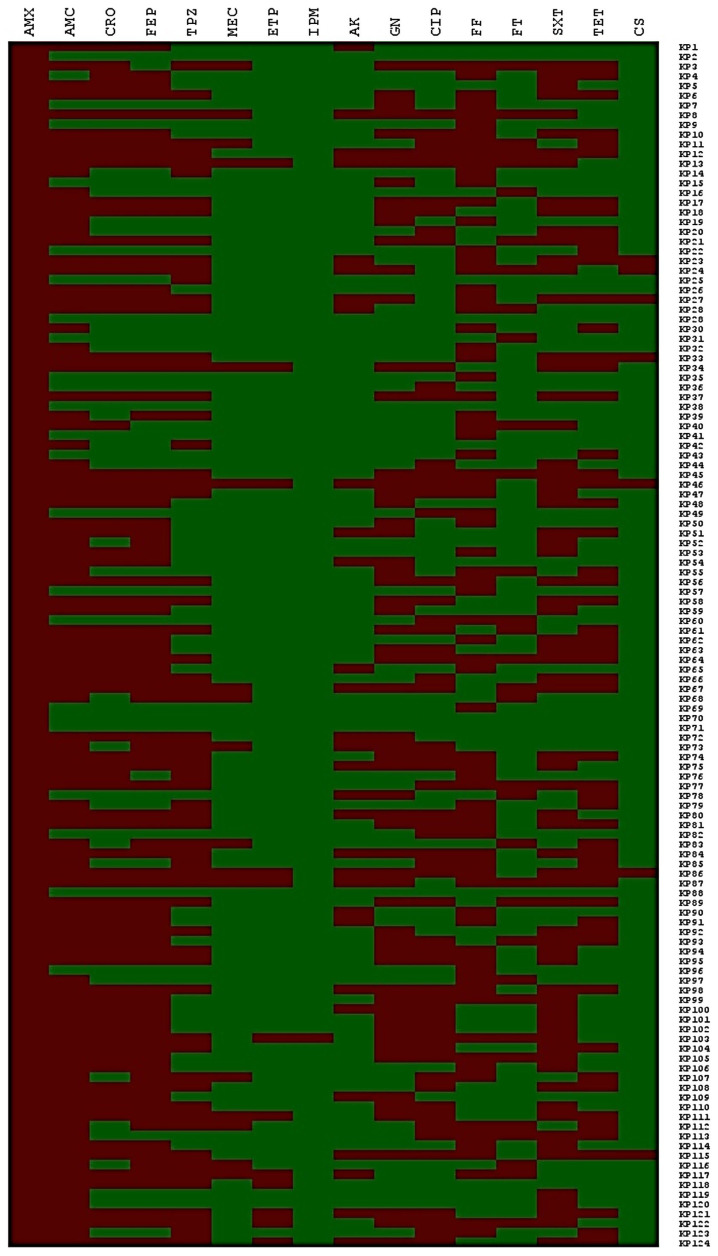
Dendrogram analysis of antibiotic profiles in *K. pneumoniae* strains: antibiotic susceptibility assessment; amoxicillin (**AMX**), amoxicillin + clavulanic acid (**AMC**), cefepime (**FEP**), piperacillin + tazobactam (**TZP**), mecillinam (**MEC**), ceftriaxone (**CRO**), ertapenem (**ETP**), imipenem (**IPM**), fosfomycin (**FF**), nitrofurantoin (**F**), trimethoprim + sulfamethoxazole (**SXT**), amikacin (**AK**), ciprofloxacin (**CIP**), tetracycline (**TET**), colistin (**CS**), gentamicin (**GN**).

The study of resistance to different antibiotics of the *K. pneumoniae* strains isolated in our study is shown in Figure 2. The resistance profile displayed the following resistance rates: amoxicillin (100%, 124/124), amoxicillin/clavulanic acid (80.6%, 100/124), mecillinam (13.7%, 17/124), piperacillin–tazobactam (51.6%, 64/124), ceftriaxone (62%, 77/124), cefepime (66.1%, 82/124), fosfomycin (59.6%, 74/124), nitrofurantoin (26.6%, 33/124), cotrimoxazole (54.8%, 68/124), ciprofloxacin (47.5%, 59/124), tetracycline (45.1%, 56/124), and aminoglycosides [amikacin (24.1%, 30/124) and gentamycin (49.1%, 61/124)]. Regarding resistance to carbapenems, 12 strains of *K. pneumoniae* were resistant to ertapenem (9.6%, 12/124), with MICs varying between 0.75 and 32 mg/L, and only one strain was resistant to imipenem (0.8%, 1/124) with an MIC equal to 32 mg/L (this strain was also resistant to ertapenem (MIC = 32 mg/L) (Table 3). We also note that most of the strains resistant to carbapenems were found in services at risk, such as ICU (41.6%), often isolated from blood (50%). All strains expressed resistance to other β-lactams (100%, 12/12), and most were resistant to other antibiotics such as gentamycin (83.3%, 10/12) and ciprofloxacin (75%, 9/12).

For colistin, seven strains of *K. pneumoniae* were resistant to this molecule (5.6%, 7/124), with MICs between 4 and 32 mg/L. These strains are isolated from pus (two cases), urine (one case), blood (one case), sputum (one case), pleural fluid (one case) and cerebrospinal fluid (one case). We also note that among the strains resistant to colistin, two strains (28.5%, 2/7) were resistant to carbapenems (KP46 and KP86) (Table 4). A comparison of resistance to colistin in carbapenemase-producing *K. pneumoniae* isolates with sensitive isolates did not show a significant difference (χ^2^ = 0.0312, *p*-value = 0.859842). 

### 3.3. Molecular Characterization of β-Lactamases, Carbapenemases, mcr Genes and mgrB Modifications

**Figure 3:**
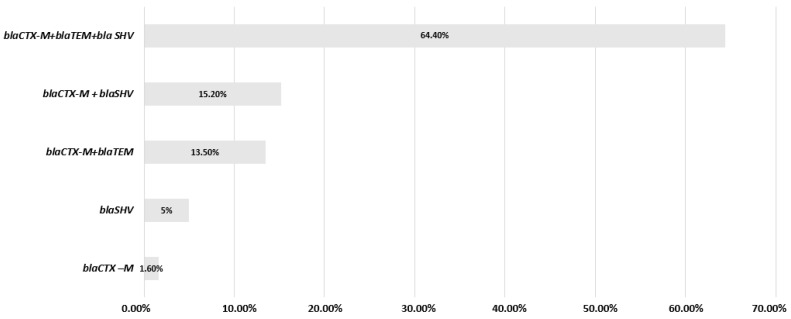
Genetic landscape: ESBL genes in *K. pneumoniae* strains.

The synergy test was positive for 59 strains, the rate of ESBL-producing isolates was 47.5% (59/124). The confirmation of phenotypic resistance to ESBL, aligning with molecular findings, indicated that PCR and subsequent sequencing analysis demonstrated the presence of the *bla*_CTX-M_ gene alone in one case (1.6%) and the *bla*_SHV_ gene alone in three cases (5%). The combination of the *bla*_CTX-M_ gene with the *bla*_TEM_ gene is found in eight strains (13.5%), and the combination of the two *bla*_CTX-M_ and *bla*_SHV_ genes is noted in nine strains (15.2%). The *bla*_CTX-M_, *bla*_TEM_ and *bla*_SHV_ association is noted in 38 strains (64.4%) (Figure 3). In sum, the three genes *bla*_CTX_, *bla*_TEM_ and *bla*_SHV_ were found in the following proportions: 94.90%, 77.90% and 84.70%, respectively. Furthermore, the Carba NP test was positive for 12 *K. pneumoniae* strains; however, the PCR and sequencing results confirmed the presence of the *bla*_OXA-48_ gene in only nine of these strains. The genes of the remaining positive strains were not demonstrated despite the positive phenotypic resistance, which may be explained by the presence of other genes not investigated in our study. The other carbapenemase types *bla*_NDM_, *bla*_IMP_ and *bla*_VIM_ were not found in our study. Among the strains resistant to carbapenems, six strains (50%, 6/12) are ESBL producers (KP13, KP34, KP46, KP86, KP87 and KP122). Concerning resistance to colistin, the confirmation of phenotypic resistance to colistin, assessed using MIC testing, aligns with the molecular findings, showing that PCR and subsequent sequencing analyses verified the presence of resistance genes. This approach enhances our understanding of how phenotypic resistance correlates with the presence of specific resistance genes. As a result, among the seven strains resistant to colistin, two strains (KP23 and KP33) harbored simultaneously the *mcr-8* gene and a mutation of the *mgr B* gene, as well as the *bla*_CTX M_, *bla*_TEM_ and *bla*_SHV_ genes, one isolated in a 48-year-old woman from sputum, the other in a little girl aged 11 months from CSF. Two other strains (KP46 and KP86) harbored a mutation in the *mgrB* gene, the *bla*_OXA-48_ gene and the *bla*_CTX M_, *bla*_TEM_ and *bla*_SHV_ genes, one in a 25-day-old child isolated from pus, the other in a 2-year-old boy isolated from urine. Three strains presented a mutation in the *mgrB* gene, as well as the coexistence of the *bla*_CTX M_, *bla*_TEM_ and *bla*_SHV_ genes (Table 2). All colistin-resistant strains carried an ESBL gene in our study.

## 4. Discussion

*Enterobacteriaceae* are the most common causes of community or nosocomial infections. They are generally treated with Beta-lactams, including penicillins, broad-spectrum cephalosporins and carbapenems, or even fluoroquinolones. Over the past two decades, there has been a significant increase in the resistance of *Enterobacteriaceae* to these antibiotics. Among *Enterobacteriaceae*, *K. pneumoniae* is also the most common MDR pathogen in healthcare settings [30]. The behavior of the strains towards antibiotics in our study showed high resistance rates towards penicillins and cephalosporins. Significant resistance rates are also observed for cotrimoxazole, aminoglycosides, tetracycline, ciprofloxacin and fosfomycin. Lower resistance rates were obtained for mecillinam (13.7%) and nitrofurantoin (26.6%). ESBL production was detected at a high rate of 47.5%. These results are in part due to the excessive and inappropriate use of antibiotics in Algeria over the last few decades.

Since the early 2000s, the overall incidence of *Enterobacteriaceae*-producing extended-spectrum β-lactamases has increased in many countries to the point where it could now constitute an ESBL pandemic [31]. ESBLs are most commonly detected in *K. pneumoniae*, which is an opportunistic pathogen associated with severe infections in hospitalized patients, including immunocompromised hosts with severe underlying diseases [32]. ESBL-producing *K. pneumoniae* was first reported in 1983 in Germany, with a steady worldwide increase in *K. pneumoniae*-mediated resistance against cephalosporins in the subsequent decades [33]. The prevalence of ESBL-producing *K. pneumoniae* varied widely and increased over time globally [34]. In Africa, for example, recent studies have reported different rates varying between 29% in South Africa and 84% in cote d’Ivoire [35,36]. In neighboring countries like Morocco, the rate of ESBL-producing *K. pneumoniae* was 35.13% [37]. In Algeria, according to a study carried out in Tizi-Ouzou, the rate of ESBL-producing *K. pneumoniae* even reached 98.8% [38]. This situation constitutes a public health problem because these bacteria, not only resistant to most β-lactams, often present resistance associated with other classes of antibiotics used in human therapy, in particular fluoroquinolones, aminoglycosides and sulfamethoxazole–trimethoprim combinations [1]. The isolates of *K. pneumoniae* obtained during this study showed a high percentage of resistance to fosfomycin and tetracycline. The results of percentage resistance in our study were significantly higher than those reported by several authors around the world concerning fosfomycin [39,40]. Almost the same rate of resistance to tetracycline was observed [41]. The acquisition of resistance to fosfomycin and tetracycline by clinical isolates of *K. pneumoniae*-sharing ESBL and/or carbapenemases represents a serious problem for the treatment of infections because these antibiotics have been utilized as a last resort for treatment.

The results of the PCR amplification of the genes *bla*_CTX-M_, *bla*_TEM_ and *bla*_SHV_ showed that almost all the strains harbored a *bla*_CTX-M_ gene (94.9%). This may explain the high level of resistance to third- and fourth-generation cephalosporins. The simultaneous presence of the three genes was found in 68.4% in our study. In the last decade, the most common ESBL gene types have been *bla*_SHV_, *bla*_TEM_ and *bla*_CTX-M_. *bla*_TEM_ and *bla*_SHV_ were the most common b-lactamase gene types, but recently, the *bla*_CTX-M_ type spread all over the world [30,42]. This type is currently most widespread in Algeria and Morocco [37,43]. The genetic variability in clinical strains, resulting from different combinations of enzyme expression, can influence the severity of infection, the response to antibiotic treatments and the transmission capacity of these resistance genes within the bacterial population. This highlights the importance of a strategic and targeted approach to control the dissemination of these enzymes and improve the available therapeutic options. Over the past decade, strains of carbapenemase-producing *Enterobacteriaceae* have emerged and become endemic in certain regions of the globe [2,44]. Carbapenems are among the rare molecules still active in cases of ESBL infections. A carbapenem resistance rate of 9.6% was observed in our study.

The prevalence of carbapenem-resistant *Enterobacteriaceae*, particularly *K. pneumoniae*, is increasing worldwide [45,46]. Higher rates between 15% and 26% have been observed in the United States and India [47,48]. The resistance rate of *K. pneumoniae* to carbapenems reached 66.3% in 2020 in Greece [49]. In contrast, Japan recorded the lowest prevalence, with only 0.13% [50]. In Africa, the prevalence of carbapenemase-producing isolates in hospital settings ranged from 2.3% to 67.7% in North Africa and from 9% to 60% in sub-Saharan Africa [4]. In Algeria, a recent study carried out in Setif presented a rate of resistance to carbapenems in *K. pneumoniae* of 7.6% [5]. The continued emergence of carbapenem-resistant *Enterobacteriaceae* constitutes a major public health threat [51]. Moreover, 75% of the strains isolated in our study harbored the *OXA-*48 gene. The resistance to carbapenems of three strains might not be related to an enzymatic mechanism such as carbapenemases, as suggested by the negative results of the amplification of the *bla*_VIM_, *bla*_IMP_, *bla*_KPC_, *bla*_NDM_ and *bla*_OXA48_ genes. It has been reported that the production of certain β-lactamases such as cephalosporinases or ESBL associated with a mechanism of impermeability by the loss of porins can lead to the carbapenem resistance of some strains of *K. pneumoniae* [52]. The production of carbapenemase constitutes the most powerful mechanism of resistance of enterobacteria to carbapenems [53]. Health authorities around the world are proposing to particularly identify carriers of enterobacteria expressing carbapenemases, which are, in a way, markers of multi-resistance to antibiotics [54,55]. Most carbapenemases confer resistance to a large number of β-lactams and their genes are usually associated with resistance genes to aminoglycosides and fluoroquinolones [56]. In our study, all carbapenemase-producing strains were resistant to amoxicillin, amoxicillin–clavulanic acid, piperacillin–tazobactam, ceftriaxone and cefepime, 88.9% were resistant to ciprofloxacin and gentamycin and 44.4% expressed ESBL. Strains resistant to carbapenems have significant epidemic power: the genes coding for carbapenemases are often located on plasmids that can be transferred from one strain to another. Currently, *bla*_KPC_, *bla*_NDM_ and *bla*_OXA-48_ are the most common carbapenemases worldwide [57]. KPCs are most commonly identified in *K. pneumoniae* in the United States, China, Colombia, Israel, Greece, and Italy, while NDMs are primarily found in *K. pneumoniae, E. coli,* and *Enterobacter spp*. from the Indian subcontinent and the *bla*_OXA-48_ type carbapenemases in *K. pneumoniae* and *E. coli* from North Africa and Turkey [44]. According to several studies, the *bla*_OXA-48_ gene, described as a class D carbapenemase, is considered an endemic gene in Algeria, first in Constantine, then in Batna, and later, in Annaba city [58,59,60]. In this study, seven strains of *K. pneumoniae* were resistant to colistin (5.6%). The global increase in MDR *K. pneumoniae* strains has increased the use of colistin to treat these infections, resulting in the emergence of colistin resistance worldwide [61]. Varying rates of resistance to colistin have been observed worldwide: 0% in Egypt [62], 0.3% in Bangladesh [63], 16% in Spain [64], 28.5% in Australia [65], and 34.5% in Saudi Arabia [6]. Another study conducted in Iran reported 98.8% colistin resistance in *K. pneumoniae* isolates [66]. Although colistin currently maintains a high activity level against most *K. pneumoniae* isolates, the decrease in activity against carbapenem-resistant isolates is worrisome. A correlation between the use of colistin to treat infections caused by carbapenem resistant and the subsequent emergence of colistin-resistant strains has also been reported. Mansour et al. in Tunisia and Narimisa et al. in Iran found a significant association between carbapenemase-producing *K. pneumoniae* isolates with colistin-resistant isolates [21,67]. Our study did not show an association between carbapenem-producing *K. pneumoniae* and increased resistance to colistin. Despite this, it will be necessary to monitor the use of colistin continually.

**Figure 4:**
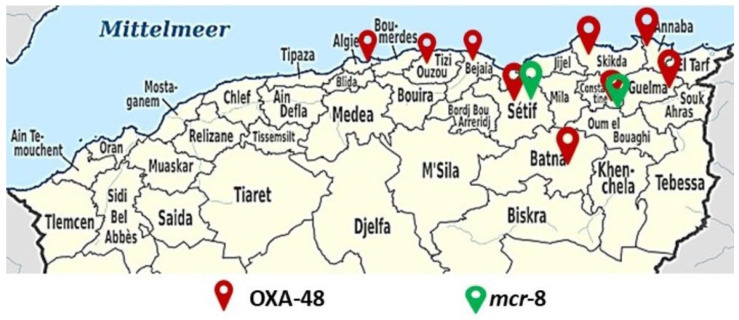
Genomic landscape: distribution of carbapenem and colistin genes in clinical isolates of *K. pneumoniae* from Northern Algeria.

All of our colistin-resistant strains harbored the mutated *mgrB* gene, of which two strains simultaneously expressed the *mcr-8* gene. Moreover, since the *mcr-1* description, eight other mobile colistin resistance genes, including *mcr*-2, *mcr-*3, *mcr-*4, *mcr*-5, *mcr-*6, *mcr-*7, and latterly, *mcr-*8 and *mcr-*9, have been reported from different countries around the world in both humans and animals [68,69,70]. In Algeria, *mcr-1, pmrAB* and *mgrB* mutations are the main reported colistin resistance mechanisms [71,72]. The first description of a clinical *K. pneumoniae* isolate harboring the *mcr-*8 gene was observed in Algeria in 2020 [11]. During our study, two strains resistant to colistin harbored the *mcr-*8 gene (KP23 and KP33), thus representing the second report in Algeria and the first in Constantine after its first description in Setif (Figure 4). In both strains, the *mcr-8* gene was associated with a mutation in the *mgrB* gene and with *bla*_CTX-M_, *bla*_TEM_ and *bla*_SHV_ (both strains harbored ESBL). This dual resistance to plasmid and chromosomal colistin, expressing high levels of resistance (MIC = 32 µg/mL) was observed for the first time, highlighting the complexity of managing resistance to this antibiotic and the need for adapted approaches to prevent and control this threat to public health. Furthermore, two strains (KP46 and KP86) were found to be positive for the *bla*_OXA-48_, *bla*_CTX-M_, and *bla*_TEM_ genes. Additionally, these strains presented an inactivating insertion in the *mgrB* gene. This represents the first report in Algeria of an association of the *bla*_OXA-48_ gene and inactivation of the *mgrB* gene. In Algeria, two reports recently revealed the presence of bacterial strains resistant to carbapenems and colistin. These strains carried *bla*_OXA-48_, *bla*_CTX-M_ and *bla*_TEM_ genes as well as a mutation in the pmrA/B gene [69,71]. The high prevalence of carbapenem and colistin-resistant strains underscores the need for revised treatment protocols and enhanced surveillance to curb the spread of these resistant pathogens.

## 5. Conclusions

Our results indicated that colistin resistance in *K. pneumoniae* isolates could be associated with a mutation in the *mgrB* gene alone or associated with the *mcr8* gene, which could increase the levels of resistance to this antibiotic. These data also provided an additional insight into the mechanism of colistin resistance. The results of our study showed a 5.6% prevalence of colistin. The prevalence of resistance to carbapenems and colistin in Algeria should be studied, and new therapeutic strategies should be evaluated. Future research should focus on multicenter studies to validate these findings and explore the development of novel therapeutic strategies. The implementation of robust infection control measures is recommended to address the growing resistance.

## Figures and Tables

**Table 1:** Primers and probes used for real-time PCR.

Gene	Primer/Probe	Sequence (5′-3′)	Size (bp)	Reference
*bla* *OXA-* *51*	OXA-51-F1 OXA-51-R1 OXA-51- Probe	GCTCGTGCTTCGACCGAGTA TTTTTGCCCGTCCCACTTAAA FAM-TCGGCCTTGAGCACCATAAGGCA-TAMRA	117	[24]
*bla*OXA-23	OXA-23-F1 OXA-23-R1 OXA-23- Probe	TGCTCTAAGCCGCGCAAATA TGACCTTTTCTCGCCCTTCC FAM-GCCCTGATCGGATTGGAGAACCA-TAMRA	130	[24]
blaOXA-24	OXA-24-F OXA-24-R OXA-24- Probe	CAAATGAGATTTTCAAATGGGATGG TCCGTCTTGCAAGCTCTTGAT FAM-GGTGAGGCAATGGCATTGTCAGCA-TAMRA	123	[24]
*bla*OXA-58	OXA-58-F OXA-58-R OXA-58- Probe	CGCAGAGGGGAGAATCGTCT TTGCCCATCTGCCTTTTCAA FAM-GGGGAATGGCTGTAGACCCGC-TAMRA	102	[24]
*bla*NDM-1	NDM-1-F NDM-1-R NDM-1-probe	GCGCAACACAGCCTGACTTT CAGCCACCAAAAGCGATGTC FAM-CAACCGCGCCCAACTTTGGC-TAMRA	155	[24]
*bla*OXA-48	OXA-48-F OXA-48-R OXA-48- Probe	TCTTAAACGGGCGAACCAAG GCGTCTGTCCATCCCACTTA FAM-AGCTTGATCGCCCTCGATTTGC-TAMRA	125	[25]
*bla* *KPC*	KPC-F KPC-R KPC-probe	GATACCACGTTCCGTCTGGA GGTCGTGTTTCCCTTTAGCC FAM-CGCGCGCCGTGACGGAAAGC-TAMRA	180	[25]
*bla VIM*	VIM-F VIM-R VIM-probe	CACAGYGGCMCTTCTCGCGGAGA GCGTACGTYGCCACYCCAGCC FAMAGTCTCCACGCACTTTCATGACCGCGTCGGC G-TAMRA	132	Jean.M. ROLAIN Laboratory [23]
*bla*CTX-M	CTX-M F CTX-M R CTX-M Probe	CGGGCRATGGCGCARAC TGCRCCGGTSGTATTGCC CCARCGGGCGCAGYTGGTGAC	105	[26]

**Table 2:** *Primers Used for Standard PCR*.

Gene	Positive Control	Primer	Sequence 5′-3′	Size (bp)	Reference
*bla*_TEM_	Kpnasey	F	ATGAGTATTCAACATTTCCGTG	861	[27]
		R	TTACCAATGCTTAATCAGTGAG		
*bla*_CTX_	Kpnasey	F	CCCATGGTTAAAAAATCACTGC	944	[26]
		R	CAGCGCTTTTGCCGTCTCCG		
*bla*_SHV_	Kpnasey	F	ATTTGTCGCTTCTTTACTCGC	1051	[28]
		R	TTTATGGCGTTACCTTTGACC		
*bla*_OXA-48_	*E. coli* CMUL64	F	TTGGTGGCATCGATTATCGG	744	[29]
		R	GAGCACTTCTTTTGTGATGGC		
*bla*_KPC_	Kpnasey	F	ATGTCACTGTATCGCCGTCT	893	Jean.M. ROLAIN Laboratory [23]
		R	TTTTCAGAGCCTTACTGCCC		

**Table 3:** Analysis of carbapenem-resistant *K. pneumoniae* strains: profiling and characteristics.

Strains	ERT MIC (mg/L)	IMP MIC (mg/L)	Carba Gene	*bla* Gene Types	Samples	Department	Age (Year)	Gender
KP13	0.75	0.25	OXA-48	*bla*_CTX-M_; *bla*_TEM_; *bla*_SHV_	Respiratory sample *	Surgery	63	M
KP34	1	2	OXA-48	*bla*_CTX-M_; *bla*_TEM_; *bla*_SHV_	Blood	ICU	19	M
KP46	0.75	0.75	OXA-48	*bla*_CTX-M_; *bla*_TEM_	Pus	Miscellaneous *	2	M
KP86	0.75	2	OXA-48	*bla*_TX-M_; *bla*_TEM_	Blood	Neonatal service	25days	M
KP87	32	2	-	*bla*_SHV_	Blood	Surgery	32	M
KP103	32	32	OXA-48		Blood	Neonatal service	23 days	F
KP111	0.75	0.5	OXA-48		Pus	Neonatal service	14 days	F
KP117	1	0.25	-		Blood	Miscellaneous **	32	F
KP118	0.75	0.38	OXA-48		Urine	ICU	43	F
KP121	1	1	OXA-48		Urine	ICU	70	M
KP122	2	0.25	-	*bla*_CTX-M_ *bla*_TEM_ *bla*_SHV_	Urine	ICU	41	F
KP124	0.5	1	OXA-48		Blood	ICU	70	M

ERT: ertapenem; IMP: imipenem; MIC: minimum inhibitory concentration; Carba R: carbapenem resistance; F: female; M: male; ICU: intensive care unit; S: susceptible; R: resistant; miscellaneous *: forensic medicine; miscellaneous **: hematology; respiratory sample *: tracheal swab.

**Table 4:** Comprehensive molecular analysis of colistin-resistant *K. pneumoniae* strains: phenotype, MIC, resistance genes, and clinical demographics.

Strains	Resistance Phenotype	Col_MIC_ (mg/L)	*bla* Gene Types	*mcr* Genes	*mgrB* Mutations	Department	Samples	Age	Gender
KP23	CT, AMX, AMC, CRO, FEP, TPZ, AK, FF, SXT, TET	16	*bla*_CTX-M_, *bla*_TEM_, *bla*_SHV_	*mcr-8*	2 insertions 1 substitution	Internal medicine	Respiratory sample *	48	F
KP24	CT, AMX, AMC, CRO, FEP, TPZ, AK GN, FF, SXT	4	*bla*_CTX-M_, *bla*_TEM_, *bla*_SHV_	*-*	1 insertion	Pediatrics	Pus	12	M
KP27	CT, AMX, AMC, CRO, FEP, TPZ, AK, GN, FF, SXT, TET	4	*bla*_CTX-M_, *bla*_TEM_, *bla*_SHV_	*-*	3 insertions	Miscellaneous *	Respiratory **	46	M
KP33	CT, AMX, AMC, CRO, FEP, TPZ, FF, SXT, TET	8	*bla*_CTX–M_, *bla*_SHV_	*mcr-8*	2 insertions	Miscellaneous *	Biological fluid *	11 months	F
KP46	CT, AMX, AMC, CRO, FEP, TPZ, MEC, ETP, AK, GN, CIP, FF, SXT, TET	16	*bla*_CTX-M_, *bla*_TEM_, *bla*_OXA-48_-	*-*	3 insertions	Neonatology	Blood	25 days	M
KP86	CT, AMX, AMC, CRO, FEP, TPZ, MEC, ETP, AK, GN, CIP, FF, SXT, TET	4	*bla*_CTX–M_, *bla*_TEM_, *bla*_OXA-48_-	-	2 insertions	Miscellaneous *	Pus	2	M
KP115	CT, AMX, AMC, CRO, FEP, TPZ, AK, GN, CIP, FF, SXT, TET	23	*bla*_CTX-M_, *bla*_TEM_, *bla*_SHV_	-	1 insertion	ICU	Urine	27	F

COL: colistin; MIC: minimum inhibitory concentration; Col R: colistin resistance; miscellenous *: forensic medicine; respiratory sample *: sputum; respiratory sample **: pleural fluid; biological fluid *: cerebrospinal fluid (CSF); F: female; M: male.

## Data Availability

The original data generated in this study are included in this article. Further enquiries can be directed to the corresponding author.
